# Suicides in Sweden

**Published:** 1860-04

**Authors:** 


*Suicide in Sweden.*—Professor Berg, of Stockholm, has recently published a report on the suicides which occurred in Sweden during the years 1843–51. They amounted to 1,308—*i. e.*, a yearly average of 145·3. A steady increase in their number has taken place since the year 1770. From 1771 to 1775 one suicide occurred amidst 65,320 inhabitants. During the first five years of the present century the proportion rose to 1 in 30,010; from 1816 to 1820, to 1 in 20,690; and from 1816 to 1850, to 1 in 14,830. In the towns the proportion is nearly three times as great as in the country districts. In Stockholm, between 1836–40, there was 1 suicide in every 5,560 inhabitants; and from 1846 to 1850, 1 in 5,390. Of 6,348 suicides occurring since 1816, 5,061 (or 79·72 per cent.) belonged to the male, and 1,287 (20·28 per cent.) to the female sex. It was seldomest committed by unmarried females, and most frequently by married men. The majority of suicides were committed between the ages of 25 and 50—viz , almost three times as many as before 25, and one-half more than after 50. By far the greatest number occurred in May, viz., more than 14 per cent.; December, November, and February furnishing the fewest numbers: 21·6 occurred in winter, 32·9 in spring, 25·9 in summer, and 20·3 in autumn. As to the form of death, hanging was resorted to in 38 per cent., and to this succeeded poisoning in 24·8 per cent., drowning in 21·4 per cent, stabbing in 8 8 per cent., and shooting in 7 per cent. In the years 1826–31, the kinds of suicide did not observe this proportion; for of 466 suicides, 126 took place by hanging, 123 by drowning, 103 by poisoning, 66 by shooting, and 48 by stabbing. Arsenic is the usual poison resorted to. Poisoning and drowning are more frequently resorted to by women than men; while shooting, hanging, and stabbing occur oftenest among men. In the various districts of the kingdom, the kinds of suicide vary much; some of these, as poisoning or shooting, being in certain parts quite unknown. Drunkenness has been juridically proved as the cause of suicide in more than 20 per cent. of the cases.—*London Medical Circular.*

